# Derivation of a Contextually-Appropriate COVID-19 Mortality Scale for Low-Resource Settings

**DOI:** 10.5334/aogh.3278

**Published:** 2021-03-26

**Authors:** J. L. Pigoga, Y. O. Omer, L. A. Wallis

**Affiliations:** 1 Division of Emergency Medicine, University of Cape Town, Cape Town, South Africa; 2 Sudan Medical Specialization Board, Khartoum, Sudan

## Abstract

**Background:**

In many low- and middle-income countries, where vaccinations will be delayed and healthcare systems are underdeveloped, the COVID-19 pandemic will continue for the foreseeable future. Mortality scales can aid frontline providers in low-resource settings (LRS) in identifying those at greatest risk of death so that limited resources can be directed towards those in greatest need and unnecessary loss of life is prevented. While many prognostication tools have been developed for, or applied to, COVID-19 patients, no tools to date have been purpose-designed for, and validated in, LRS.

**Objectives:**

This study aimed to develop a pragmatic tool to assist LRS frontline providers in evaluating in-hospital mortality risk using only easy-to-obtain demographic and clinical inputs.

**Methods:**

Machine learning was used on data from a retrospective cohort of Sudanese COVID-19 patients at two government referral hospitals to derive contextually appropriate mortality indices for COVID-19, which were then assessed by C-indices.

**Findings:**

Data from 467 patients were used to derive two versions of the AFEM COVID-19 Mortality Scale (AFEM-CMS), which evaluates in-hospital mortality risk using demographic and clinical inputs that are readily obtainable in hospital receiving areas. Both versions of the tool include age, sex, number of comorbidities, Glasgow Coma Scale, respiratory rate, and systolic blood pressure; in settings *with* pulse oximetry, oxygen saturation is included and in settings *without* access, heart rate is included. The AFEM-CMS showed good discrimination: the model including pulse oximetry had a C-statistic of 0.775 (95% CI: 0.737–0.813) and the model excluding it had a C-statistic of 0.719 (95% CI: 0.678–0.760).

**Conclusions:**

In the face of an enduring pandemic in many LRS, the AFEM-CMS serves as a practical solution to aid frontline providers in effectively allocating healthcare resources. The tool’s generalisability is likely narrow outside of similar extremely LRS settings, and further validation studies are essential prior to broader use.

## Introduction

Initial reports suggested that low- and middle-income countries (LMICs), whose health systems are generally categorised as underdeveloped [1], would face the brunt of COVID-19 cases and fatalities [2,3]; however, present data suggest that these concerns may have been premature. When compared to their high-income country (HIC) counterparts, many African LMICs have fared well, with substantially fewer recorded infections [4]. Some of this difference may be accounted for in underreporting, because LMICs are more likely to have limitations in testing and reporting capacities [5,6]. Younger populations and pre-existing immunity due to overall higher rates of infectious diseases, including other coronaviruses, may also have contributed to lower rates of infection [7]. It is also likely that the swift implementation and strong enforcement of effective public health practices, such as lockdowns, along with previous experiences in responding to other outbreaks, aided these countries in stemming the early spread of COVID-19 disease [8]. While employing public health tactics proved effective in the medium-term, buying these health systems valuable time to build capacity for larger responses, these strategies have resounding social, economic, and health-related impacts and are not sustainable in the longer term [9]. As lockdowns are loosened around the globe, these regions have experienced an uptick in cases [10].

In LMICs, only 20% of the population is forecasted to receive vaccination against COVID-19 this calendar year [11], and cases are likely to remain high. This lack of vaccinations, in combination with previously low infection rates and nascent healthcare systems, means that the pandemic may continue for the foreseeable future in African LMICs [12]. Clinicians and health system planners need to be able to allocate limited healthcare resources as cases inevitably rise. Establishing an early understanding of a patient’s likely outcome is integral to decreasing mortality in LRS, with previous evidence suggesting that risk stratification can improve patient outcomes from a range of illnesses and injuries, while reducing resource utilisation [13,14]. While many prognostication tools have been developed for, or applied to, COVID-19 patients, no tools to date have been purpose-designed for, and validated in, truly low-resource settings (LRS). A recent review identified 39 risk stratification scores (including severity and mortality indices) being used for COVID-19 prognostication. Only two of these tools were deemed feasible for use in LRS, where laboratory and imaging are not readily available, and neither was intended for such a setting or validated in it [15,16,17,18]. The enduring impacts of the COVID-19 pandemic on LMICs make it essential that clinical decision-making tools with the potential to reduce mortality be made widely available. A mortality scale holds promise in aiding frontline providers in LRS in managing the pandemic SARS-COV-2 infection and COVID-19 disease by allowing them to allocate potential resources that a patient will need throughout the duration of both emergency unit (EU) and hospital stays based on mortality likelihood.

This study aimed to develop a pragmatic tool to assist facility-based healthcare providers in LRS in evaluating in-hospital mortality risk using only demographic and clinical inputs that are readily obtainable upon arrival in EUs.

## Methods

Machine learning was used on data from a retrospective cohort of Sudanese COVID-19 patients to derive a contextually appropriate mortality scale for COVID-19. Tool derivation and reporting followed the Transparent Reporting of a Multivariable Prediction Model for Individual Prediction or Diagnosis (TRIPOD) guidelines [19].

### Study setting

The Republic of the Sudan, Africa’s third largest nation, is home to 41.8 million [20]. The country’s population is young, with 80% of citizens under the age of 40 and an average life expectancy at birth of 64 years [20,21]. Sudan’s health system is strained by a number of causes of morbidity and mortality, including road traffic injuries and cardiovascular conditions, and there are also persistent challenges in maternal and child mortality [20,21]. The public health system is decentralised across three levels—federal, state, and local—and there are two levels of hospitals, known as district and referral. District hospitals provide lower-level care and tend to be located in rural and peri-urban areas, while referral hospitals typically offer more advanced care in urban centres. Throughout the COVID-19 pandemic, however, these facilities have been providing similar levels of care, regardless of their designation as a district or referral hospital. Data for this study were obtained from two government referral hospitals in Sudan’s most populous region, Khartoum State.

### Data collection

Existing resource constraints have delayed implementation of an electronic COVID-19 patient registry in Sudan, and most facilities continue to rely on traditional paper-based charting systems. For the purposes of this study, on-site data collectors retrospectively logged deidentified historical and clinical information for patients presenting to the two study sites from paper records into a secure electronic Microsoft Excel (© Microsoft, Redmond, WA, USA) database stored locally. Data were randomly split into two cohorts—80% for training and 20% for testing—for derivation purposes.

### Study population

All patients were adults 18 years and over who presented to study sites between 01 April to 01 September 2020; met WHO criteria for a suspected, probable, or confirmed case of SARS-CoV-2 infection [22]; and had a recorded disposition of either death or discharge.

### Primary outcome

The primary outcome of this study was in-hospital mortality due to SARS-CoV-2 infection.

### Candidate predictor variables

A set of potentially predictive variables was defined *a priori* based on a review of validation data for previously designed pneumonia and sepsis scores, and purpose-designed COVID-19 indices, in COVID-19 populations (Appendix 1). This list was generated in the context of the results of two studies—a scoping review and systematic review and meta-analysis—that evaluated the feasibility and significance of a range of variables in predicting severe COVID-19 in LRS [15,23]. These potential candidate predictor variables comprise demographic and historical data, presenting signs and symptoms, and vital signs and associated parameters consistently identified to be clinically important in COVID-19 cohorts. In order to be included in model development, a potential predictor variable needed to be included in a risk stratification tool validated in a COVID-19 population or evidenced as an independent predictor of COVID-19 severity.

Available literature suggests that demographics, comorbidities, signs and symptoms, vital signs, and blood glucose testing can feasibly be collected in most LRS EUs, while laboratory investigations and imaging generally are not [16,17,18]. Peripheral oxygen saturation—highlighted in many studies to be a key indicator of poor outcomes in COVID-19 [24,25] —has mixed but increasing availability [16,26,27]. In line with this study’s aim to develop a mortality scale that is widely applicable across LRS, no laboratory and imaging data were considered for inclusion in this scale. Although pulse oximetry is limited in some LRS, it is available in Sudanese referral hospitals, and local stakeholders requested its consideration for inclusion in this scale. As such, an *a priori* decision was made to include peripheral oxygen saturation in model development but, should it be included in the final scale, to use identical methods to develop a second scale using identical methodology that does not consider this as a potential input.

Where evidence was unavailable to support inclusion, variables were excluded. Variables with greater than 33% missingness were also excluded. Comorbidities were collapsed into clinically relevant categories by local clinicians. These categories were viewed as binary, with patients either having one or more of the included comorbidities, or none.

Given the limited size of this dataset, we elected to use the following predefined parameters for continuous predictors included in the final model (***Table 1***).

**Table 1:** Predefined parameters for continuous candidate predictor variables.


CANDIDATE PREDICTOR VARIABLE	PARAMETER(S)

Age	≥65 years [28]

Number of comorbidities	0, 1, ≥2 [29]

Glasgow Coma Scale	<15 [29,30]

Systolic blood pressure	≤100 mmHg [12]

Respiratory rate	20–29 breaths/min, ≥30 breaths/min [29]

Heart rate	>90 beats/min [31]

Peripheral oxygen saturation on room air	<92% [29]

Temperature	>38°C or <36°C [31]


### Data missingness

Multiple imputation by chained equations (MICE) was used for predictors in instances where less than 33% of data points were missing. Data were assumed to be missing completely at random. A total of 10 iterations of MICE were conducted, with predictive mean matching as the designated method for continuous variables and binary logistic regression for two-level variables. Distributions of missing and observed data were reviewed for each predictor, and Rubin’s rules were used to combine results [32].

### Model development

A criterion-based method of model selection, similar to that used by Knight and colleagues [29], was utilised to build a model for predicting in-hospital mortality risk due to COVID-19. Generalised additive models (GAMs) were used to assess the deviance explained by independent predictors, with variables generating >1% change in deviance explained included in the final model.

Least absolute shrinkage and selection operator (LASSO) logistic regression on a multiply-imputed dataset was used for coefficient estimation. LASSO was selected to prevent overfitting due to the large number of predictor variables. A 10-fold cross-validation was performed to identify the optimal value of lambda, from which L1 penalised coefficients were generated. These L1 penalised coefficients were then proportionally scaled to create a usable scoring scale for the AFEM-CMS. Scatter plots distributions comparing scores and case fatality rates were visually inspected by local clinicians to determine clinically meaningful cut-off points for risk groups.

Model performance in the derivation dataset was assessed using the C-index (area under the receiver operator curve (AUROC)) in the testing dataset.

All analyses were performed in R (version 4.0.2, © The R Foundation) using the *dplyr, finalfit, glmnet, mice, pROC, rmda*, and *tidyverse* packages.

## Results

### Characteristics of study population

A total of 467 patients met the inclusion criteria and were included in this study (***Table 2***). Only one third of the cohort (n = 177) had recorded results for polymerase chain reaction (PCR) testing, of which 75.1% were positive. All remaining patients were diagnosed via chest CT scan. All patients met the WHO case definition for suspected, probable, or confirmed SARS-CoV-2 infection [22]. The overall case fatality rate for these admitted patients was 51.2%. Survivors had a median age of 61 years (interquartile range (IQR): 21.3 years); this was slightly younger than non-survivors’ median age of 70 (IQR: 16) years. A majority of patients—63.4% of survivors (n = 149) and 74.9% of non-survivors (n = 179)—were male. More than three quarters of patients (n = 341, 77.3%) had at least one comorbidity. The most prevalent comorbidities in these cohorts were hypertension (present in 36.2% of survivors and 57.4% of non-survivors) and diabetes (33.9% of survivors and 52.0 (n = 116) of non-survivors). Shortness of breath was the most common self-reported symptom, seen in 68.0% of patients, followed by cough (34.5%) and fever (30.7%). Median Glasgow Coma Scale (GCS), peripheral oxygen saturations on room air, and systolic blood pressures were lower in the non-survivor cohort when compared to survivors, while heart and respiratory rates and temperatures were higher in this group.

**Table 2:** Characteristics of study population.


CHARACTERISTIC	OVERALL COHORT (N = 467)		SURVIVOR COHORT (N = 228)		NON-SURVIVOR COHORT (N = 239)	

	n (%) OR MEDIAN (IQR)	TOTAL NO PATIENTS (%)	n (%) OR MEDIAN (IQR)	TOTAL NO PATIENTS (%)	n (%) OR MEDIAN (IQR)	TOTAL NO PATIENTS (%)

In-hospital mortality	239 (51.2)	467 (100.0)	0 (0.0)	228 (100.0)	239 (100.0)	239 (100.0)

Positive PCR testing result	133 (75.1%)	177 (37.9)	74 (81.3)	91 (39.9)	59 (68.6)	86 (40.0)

**DEMOGRAPHICS**						

Age (years)	65 (19)	467 (100.0)	61 (21.3)	228 (100.0)	70 (16)	239 (100.0)

Male sex at birth	328 (70.2)		149 (63.4)	–	179 (74.9)	–

COMORBIDITIES*						

Alcohol use	10 (2.3)	441 (21.8)	5 (2.3)	218 (95.6)	5 (2.2)	223 (93.3)

Cardiovascular disease	50 (11.3)	–	26 (11.9)	–	24 (10.8)	–

Chronic respiratory disease	39 (8.8)	–	19 (8.7)	–	20 (9.0)	–

Rheumatic/connective tissue disease	4 (0.9)	–	1 (0.4)	–	3 (1.3)	–

Chronic neurological disease	32 (7.3)	–	14 (6.4)	–	18 (8.1)	–

Cirrhosis	5 (1.1)	–	3 (1.4)	–	2 (0.9)	–

Chronic kidney disease	34 (7.7)	–	17 (7.8)	–	17 (7.6)	–

Diabetes (type 1 and 2)	190 (43.1)	–	74 (33.9)	–	116 (52.0)	–

Hypertension	207 (49.6)	–	79 (36.2)	–	128 (57.4)	–

Hypothyroid	3 (0.7)	–	2 (0.9)	–	1 (0.4)	–

Malignancy	16 (3.6)	–	5 (2.3)	–	11 (4.9)	–

Current or former smoker	30 (6.8)	–	10 (4.6)	–	20 (9.0)	–

Current pregnancy**	1 (0.7)	82 (59.0)	0 (0.0)	37 (46.8)	1 (1.6)	45 (75.0)

Total no. comorbidities						

0	100 (22.7)	441 (94.4)	66 (30.3)	218 (95.6)	34 (15.2)	223 (93.3)

1	150 (34.0)	–	80 (36.7)	–	70 (31.4)	–

≥2	191 (43.3)	–	72 (33.0)	–	119 (53.4)	–

**PRESENTING SIGNS & SYMPTOMS**						

Abdominal pain and/or distention	12 (3.0)	394 (84.4)	10 (5.1)	196 (86.0)	2 (1.0)	198 (82.8)

Anorexia, nausea, and/or vomiting	33 (8.4)	–	18 (9.2)	–	15 (7.6)	–

Chest pain or tightness	11 (2.8)	–	5 (2.6)	–	6 (3.0)	–

Convulsions	4 (1.0)	–	1 (0.5)		3 (1.5)	–

Cough	136 (34.5)	–	66 (33.7)	–	70 (35.4)	–

Diarrhoea	12 (3.0)	–	7 (3.6)	–	5 (2.5)	–

Fatigue	58 (14.7)	–	31 (15.8)	–	27 (13.6)	–

Fever	121 (30.7)	–	67 (34.2)	–	54 (27.3)	–

Headache	34 (8.6)		19 (9.7)	–	15 (17.6)	–

Internal bleeding***	9 (2.3)	–	4 (2.0)	–	5 (2.5)	–

Myalgia	10 (2.5)	–	5 (2.6)	–	5 (2.5)	–

Shortness of breath	268 (68.0)	–	125 (63.8)	–	143 (72.2)	–

Sore throat	25 (6.3)	–	14 (7.1)	–	11 (5.6)	–

**VITAL SIGNS**						

Glasgow Coma Scale score	15 (1.0)	375 (80.3)	15 (0.0)	179 (78.5)	14 (3.3)	196 (82.0)

Heart rate (beat/min)	96 (24.0)	375 (80.3)	93 (21.0)	195 (85.5)	100.0 (22.0)	180 (75.3)

Peripheral oxygen saturation (%)****	91 (11.3)	372 (79.7)	94 (7.0)	186 (81.6)	87 (17.0)	186 (77.8)

Systolic blood pressure (mmHg)	130 (29.5)	367 (78.6)	131 (24.8)	195 (85.5)	123.5 (39.0)	173 (72.4)

Respiratory rate (breaths/min)	28 (12.0)	409 (87.6)	25 (9.0)	194 (85.1)	30 (14.5)	215 (90.0)

Temperature (°C)	36.9 (1.5)	62 (13.3)	36.6 (1.4)	27 (11.8)	36.9 (1.6)	35 (14.6)


PCR: Polymerase chain reaction * Some comorbidities have been collapsed into the following clinically meaningful categories: Cardiovascular disease includes one or more of the following: atrial fibrillation, congestive heart failure, coronary artery disease, deep vein thrombosis, dilated cardiomyopathy, ischaemic heart disease, myocardial infarction, and small vessel disease. Chronic respiratory disease includes one or more of the following: asthma, chronic obstructive pulmonary disease, and tuberculosis. Rheumatic/connective tissue disease includes one or more of the following: gout, lupus, and rheumatoid arthritis. Chronic neurological disease includes one or more of the following: epilepsy, haemorrhagic or ischaemic stroke, and Parkinson’s disease. ** Considered in female subpopulation only (n = 139). *** Internal bleeding includes one or more of the following: gastrointestinal bleeding, haematuria, or haemoptysis. **** Peripheral oxygen saturation obtained on room air.

### Case fatality rates

**Figure 1:**
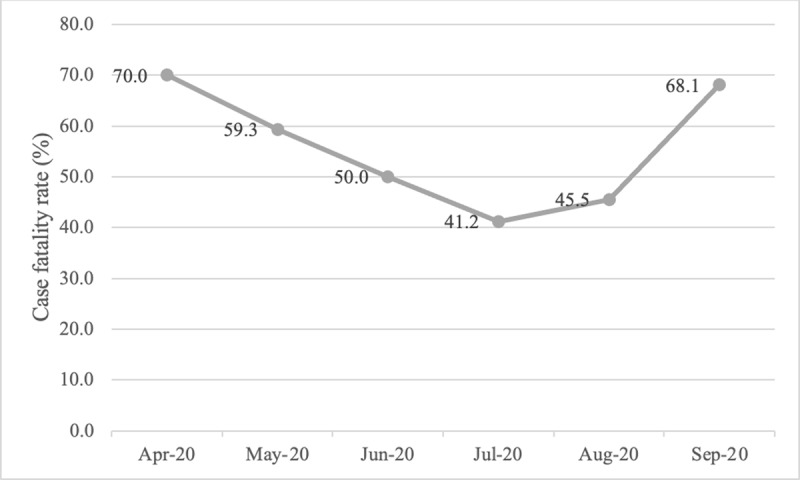
COVID-19 case fatality rates at two government referral hospitals in Sudan from April to September 2020.

Case fatality rates varied over time, with peaks near 70% in April and September 2020 and a low point of 41.2% in July 2020 (***Figure 1***).

### Model development

This record review identified 34 potential predictors of mortality measurable in LRS EUs. Some data were missing for all predictors except sex at birth, age, and final disposition. Current pregnancy and temperature were excluded from model development due to excessive missingness (missing in 41.0% and 86.7% of cases, respectively). An additional five presenting signs and symptoms and one comorbidity were removed due to lack of evidence supporting inclusion in a COVID-19 risk stratification tool. Comorbidities were collapsed into a composite variable reflecting the patient’s total number of comorbidities. Inclusion decisions for model fitting, including *a priori* evidence and data missingness, are described in detail in Appendix 1.

Seventeen variables were included in the model fitting process, which used GAMs on the multiply imputed datasets to identify significant independent predictors of COVID-19 mortality. Male sex at birth, age, number of comorbidities, total number of comorbidities, GCS, systolic blood pressure, respiratory rate, and peripheral oxygen saturation on room air were found to be predictive (Appendix 2). LASSO regression was used to generate a usable scoring system, with all variables retained in the model (Appendix 3 and ***Table 3a***).

**Table 3a:** AFEM COVID-19 Mortality Scale (AFEM-CMS) for in-hospital mortality due to COVID-19 in low-resource settings *with* access to pulse oximetry.


VARIABLE	SCORE

Sex at birth	

Female	0

Male	1

Age (years)	

<65	0

≥65	1

No. of comorbidities*	

<2	0

≥2	1

Glasgow Coma Scale	

15	0

<15	2

Systolic blood pressure (mmHg)	

>100	0

≤100	1

Respiratory rate (breaths/min)	

<20	0

≥20	1

Peripheral oxygen saturation on room air (%)	

≥92	0

<92	2


* Comorbidities are defined as follows: Alcohol use, cardiovascular disease (one or more of the following: atrial fibrillation, congestive heart failure, coronary artery disease, deep vein thrombosis, dilated cardiomyopathy, ischaemic heart disease, myocardial infarction, and small vessel disease), chronic respiratory disease (one or more of the following: asthma, chronic obstructive pulmonary disease, and tuberculosis), chronic neurological disease (one or more of the following: epilepsy, haemorrhagic or ischaemic stroke, and Parkinson’s disease), cirrhosis, chronic kidney disease, current or former smoker status, diabetes (types 1 and 2), hypertension, hypothyroid, and malignancy.

In line with this study’s aim to generate a flexible but pragmatic tool for LRS, a second analysis was run excluding peripheral oxygen saturation as a predictor. In this analysis, seven variables—male sex at birth, age, number of comorbidities, total number of comorbidities, GCS, systolic blood pressure, respiratory rate, and heart rate—were identified as important predictors of mortality (Appendix 2) and included in a second scale that is not reliant on pulse oximetry (Appendix 3 and ***Table 3b***).

**Table 3b:** AFEM COVID-19 Mortality Scale (AFEM-CMS) for in-hospital mortality due to COVID-19 in low-resource settings *without* access to pulse oximetry.


VARIABLE	SCORE

Sex at birth	

Female	0

Male	1

Age (years)	

<65	0

≥65	1

No. of comorbidities*	

<2	0

≥2	1

Glasgow Coma Scale score	

15	0

<15	2

Systolic blood pressure (mmHg)	

>100	0

≤100	2

Respiratory rate (breaths/min)	

<20	0

≥20	1

Heart rate (beats/min)	

≤90	0

>90	1


* Comorbidities are defined as follows: Alcohol use, cardiovascular disease (one or more of the following: atrial fibrillation, congestive heart failure, coronary artery disease, deep vein thrombosis, dilated cardiomyopathy, ischaemic heart disease, myocardial infarction, and small vessel disease), chronic respiratory disease (one or more of the following: asthma, chronic obstructive pulmonary disease, and tuberculosis), chronic neurological disease (one or more of the following: epilepsy, haemorrhagic or ischaemic stroke, and Parkinson’s disease), cirrhosis, chronic kidney disease, current or former smoker status, diabetes (types 1 and 2), hypertension, hypothyroid, and malignancy.

Analysis of scatter plots distributions comparing mortality scale scores and case fatality rates (Appendix 4) led to the designation of clinically meaningful cut-off points that categorised patients into three mortality risk groups, as defined in ***Table 4***.

**Table 4:** Mortality risk stratification based on AFEM COVID-19 Mortality Scale (AFEM-CMS) scores.


MORTALITY RISK	AFEM-CMS SCORE

<33%	0 to 2

33% to 66%	3 to 5

>66%	6 to 9


### Model assessment

In both versions of the scale, most patients—47.3% (n = 221) in the tool with SPO2 and 57.2% (n = 267) in the one without—were estimated to have between 33% and 66% risk of death. Distributions of scores are shown in ***Table 5*** and ***Figures 2a*** and ***2b***.

**Table 5:** Comparison of mortality risk rates by mortality risk group for 467 COVID-19 patients used to train the AFEM COVID-19 Mortality Scale (AFEM-CMS).


MORTALITY RISK GROUP	SCORE	NO SPO2 SCORE			SPO2 SCORE		

		NO. PATIENTS	NO. FATALITIES	CFR (%)	NO. PATIENTS	NO. FATALITIES	CFR (%)

<33%	0 to 2	68	5	7.4	84	5	6.0

33% to 66%	3 to 5	267	124	46.4	221	103	46.6

>66%	6 +	132	110	83.3	162	131	80.9


* CFR: Case fatality rate.

**Figure 2a:**
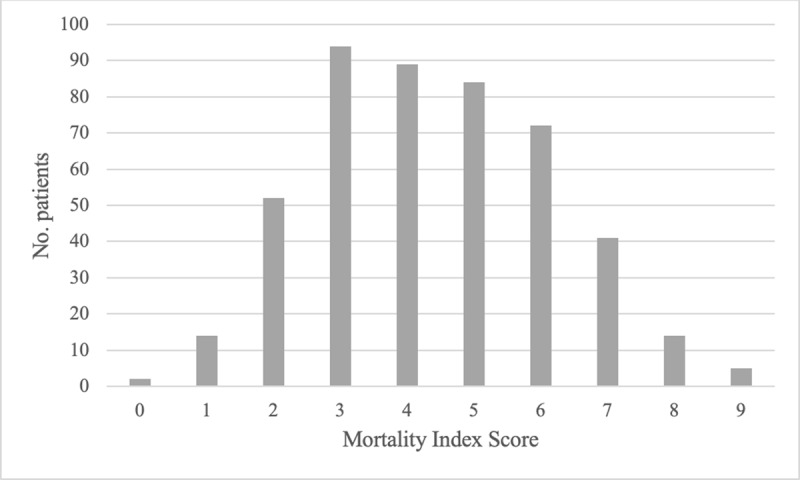
Distribution of patients across AFEM COVID-19 Mortality Scale (AFEM-CMS) scores in derivation cohort, for resource settings with access to pulse oximetry.

**Figure 2b:**
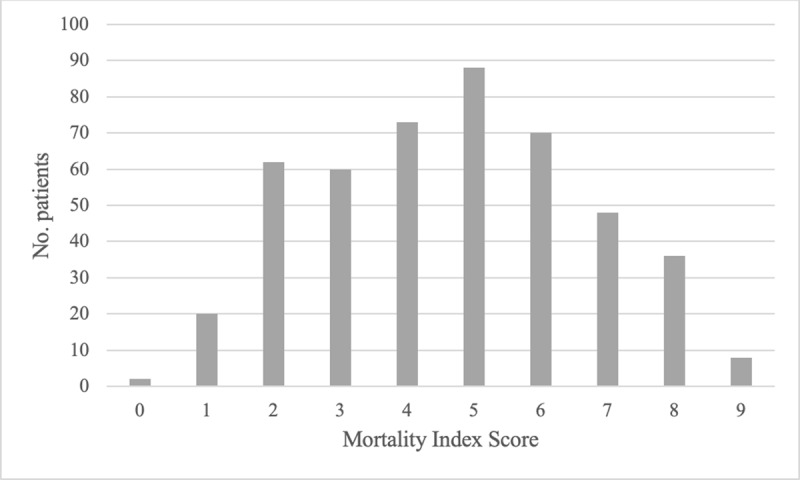
Distribution of patients across AFEM COVID-19 Mortality Scale (AFEM-CMS) scores in derivation cohort, for resource settings without access to pulse oximetry.

Both versions of the mortality scale showed good discrimination. The model including pulse oximetry had a C-statistic of 0.780 (95% CI: 0.740–0.820) in the training set and 0.775 (95% CI: 0.737–0.813) in the testing set. For the model excluding pulse oximetry, the C-statistic was 0.732 (95% CI: 0.686–0.778) in the training set and 0.719 (95% CI: 0.678–0.760) in the testing set.

## Discussion

This study led to the successful generation of the AFEM-CMS, a pragmatic mortality scale purpose-designed for frontline clinicians in extremely LRS. Two versions of the tool exist, each using seven demographic, historical, and clinical inputs to evaluate potential risk of death in COVID-19 patients. The tool is intended for frontline use and, as such, does not include clinical inputs that may take significant time to obtain or may be completely unavailable in LRS EUs, such as laboratory and imaging investigations. One version of the AFEM-CMS does, however, include pulse oximetry, which has been found to be a strong predictor of poor outcomes in COVID-19 patients but has variable availability in LMICs [16,24,25,26,27]. Both versions of the tool have good discriminatory power, although associated confidence intervals are somewhat wide. While the C-statistic for the scale including oxygen saturation is somewhat higher than for the one excluding it, their confidence intervals do overlap. Given this, it is difficult to state with certainty that the model including oxygen saturation is better and should be prioritised for use in whenever possible. Some settings may have the capacity to perform pulse oximetry but have a limited number of monitors to do so; in these instances, it may be detrimental to slow down the assessment process in efforts to obtain a patient’s oxygen saturation for use with the AFEM-CMS. It will be crucial that recommendations for AFEM-CMS implementation address such issues, and we emphasise that current data do not suggest significantly poorer discrimination in the tool without oxygen saturation.

Data from 34 independent predictors were available; however, two of these were not included in model fitting due to excessive missing data points and another six due to lack of evidence that they are predictive of mortality in COVID-19 disease. Presenting signs and symptoms accounted for 8 of the 17 variables considered in our model. Despite evidence suggesting that these variables may be predictive of severe disease, no signs or symptoms were included in the final model. The case was the same for smoking, which has been identified in multiple meta-analyses as predictive of disease progression.

The AFEM-CMS shares many similarities with existing risk stratification tools for COVID-19 but is unique in its combination of variables and weightings. Similar to many other COVID-19 specific tools, such as A-DROP [33] and the 4C Mortality Score, [33] and pneumonia severity indices, such as PSI [34], both age and male sex at birth were identified as significant predictors of in-hospital mortality due to COVID-19 in both versions of the AFEM-CMS. Comorbidities were also a key predictor of mortality in both models. But unlike in the 4C Mortality Score, from which our parameters for this variable—the presence of 0, 1, or ≥2 comorbidities—were chosen, mortality was only predicted by the presence of two or more comorbidities; one comorbidity was only slightly more predictive than zero.

In the version of the AFEM-CMS including pulse oximetry measurements, all vital signs except heart rate were included in the final model. In the absence of oxygen saturation measurements, however, heart rate became significantly predictive. Heart rate is likely serving as a proxy for decreased oxygenation in the model without pulse oximetry, as it will typically increase to compensate for decreasing blood oxygen levels. Three other vital signs are included in both models: systolic blood pressure, GCS, and respiratory rate. Decreases in systolic blood pressure and consciousness are late indicators of clinical deterioration and indicate increased potential for mortality. Respiratory rate has been characterised as a key predictor in nearly all existing COVID-19 risk stratification scores, and although cut-points for significance have varied, most tools consider rates greater than approximately 20 breaths per minute as abnormal. We originally sought to stratify patients into three classes—<20, 20–29, and ≥30—as is seen in the 4C Mortality Score; however, the penalised coefficients for the 20–29 and ≥30 classes were extremely similar and generated the same weighting in both models. This led to our tools having only one cut-point for respiratory rate.

We sought to generate a scale that categorised patients into one of three clinically meaningful levels of mortality risk. Most three-level mortality indices for COVID-19 and pneumonia described in the literature use standardised mortality-risk cut points to classify patient severity [28,29,35,36]. For example, in the COVID-19-specific 4C Mortality Score, a mortality rate below 2% represents low risk, a rate between 2% and 14.9% represents intermediate risk, and a rate 15% and above represents high risk [29]. Our original intention was to align our scale with these widely used cut points; however, analysis of the distribution of fatalities across AFEM-CMS scores suggested that case fatality rates (CFRs) for COVID-19-patients in Sudanese hospitals were much too high for the aforementioned cut-point values to have any clinical utility. As such, substantially higher but much more clinically relevant cut points were selected that categorise patients into mortality risks of below 33%, 33% to 66%, and above 66%.

The Sudanese population was facing healthcare and political crises even before the pandemic, and COVID-19 has only worsened health and economic statuses [5,37]. The healthcare system is vulnerable, with limited capacity to respond to the COVID-19 surge [38]. Calls have been made for unique, tailor-made approaches to managing the pandemic in Sudan [37], but it is difficult to respond to such requests without data to inform potential solutions. This study, which represents the largest record review of Sudanese COVID-19 patients admitted to hospitals in the literature to-date, provides key information about hospitalised patients in the country that may be of use to researchers and systems planners. It describes an extremely ill cohort of patients: A majority have at least one comorbidity, the median age is higher than the nation’s general life expectancy, and most presented with some abnormal vital signs. More than half of this cohort died in-hospital. Unfortunately, it is expected that patients presenting to hospitals in Sudan with COVID-19 are very sick and at high risk of death. While mortality rates have varied widely, both geographically and temporally, in Sudan, the country has been noted to have one of the highest infection fatality rates in the Arab world: 5.7% [6]. Sudan has some of the poorest physical access to healthcare for adults in sub-Saharan Africa, with 40.9% of adults 60 years or older traveling longer than six hours to the nearest hospital [39]. Given that this age demographic comprises the bulk of those with severe COVID-19 disease, it is unsurprising that they are not able to reach definitive care until their illness has worsened substantially.

Data from this study build upon, and largely align with, the one previous cohort study conducted on COVID-19 patients in Sudan. Omar and colleagues evaluated 88 COVID-19 patients at a Sudanese government hospital in Eastern Sudan between April and July 2020 [40]. As in our study, the majority of the cohort was male, and non-survivors tended to be older than their survivor counterparts. In both studies, cough, fever, and shortness of breath were the most comment presenting signs and symptoms. Their work also identifies that age and peripheral oxygen saturation on room air at time of admission were significantly associated with in-hospital mortality, although only one comorbidity—diabetes mellitus—was found to be predictive. The in-hospital CFR in this study was slightly lower, at 37.5%, though this may be attributable to differences in geographic and resource constraints.

## Limitations

There are several limitations to this study, the most important of which is the generalisability of its results. CFRs varied over time throughout this study, with an initial decrease followed by an increase to the original rate. The AFEM-CMS was developed based on the average of these rates and may perform differently when CFRs are consistently higher or lower. Additionally, given the urgency of the pandemic, only a small dataset could be obtained from just two facilities that had the capacity to participate in this work. These patients were extremely ill and tended to be older than Sudan’s general population. As such, the AFEM-CMS’s generalisability is likely very limited outside of similar extremely LRS settings, such as those nations listed on the Development Assistance Committee’s list of Least Developed Countries [41], and its broader use cannot be recommended until additional validation work has been undertaken.

There is also potential that use of these two facilities’ datasets may have inflated CFRs to rates higher than seen more broadly in Sudan, which are reported to be closer to the rates reported by Omar and colleagues. The hospitals included in this study are referral hospitals, whose advanced care attracts sicker patients whose infections may be more likely to be fatal. To evaluate this further, future validation work will also include data from lower-level facilities.

All patients included in this study met the WHO definitions of suspected, probable, or confirmed COVID-19 infection; however, only one third of patients received PCR testing to confirm infection. Although this deficit in testing aligns with previously described testing challenges in LMICs [5,6], it does limit the results of this study. More than half of patients were diagnosed with COVID-19 using only WHO clinical and epidemiologic criteria [22], and, without laboratory confirmation, it cannot be known that all of these patients were actually infected with SARS-CoV-2. The decision to include patients meeting all levels of the WHO COVID-19 case definition was made due to the urgent need for a reasonable volume of data to inform the response.

Some self-reported information, including age, specific comorbidities, and presenting signs and symptoms, may have gone undocumented, especially in instances where patients were very ill at the time of presentation. In the Sudanese setting, where paper records are used at government facilities, it is nearly impossible to link to patients’ prior medical records to obtain historical information, and clinicians must rely solely on the narrative interview data gathered at the time of information. This information was missing at slightly higher rates in the non-survivor cohort but does not appear to be extremely different than in the survivor cohort, suggesting that such scenarios were uncommon and would not significantly influence results.

Development of the AFEM-CMS may also be limited by the use of predefined parameters for continuous clinical measurements. Although these cut-off points have been established and validated across a number of other risk stratification tools, including some for COVID-19, they may not be reliable for use in LRS. In future studies, analysis of the local distribution of these parameters will be undertaken to ensure that the current cut-off points are ideal.

## Conclusion

To our knowledge, the AFEM-CMS is the first purpose-designed tool for mortality prediction of COVID-19 patients by frontline providers in extremely LRS. It is intended for rapid use at the frontlines of the response in EUs and other receiving areas of healthcare facilities where laboratory and imaging investigations are limited or unavailable. In the face of an enduring pandemic in many LMICs, the tool could serve as a practical solution to aid frontline providers in effectively allocating limited healthcare resources. However, the tool’s generalisability is likely limited outside of similar LRS with similarly high fatality rates. Future work will be undertaken to validate the tool and to compare it to other scales that may be feasible in LRS as more data become available.

## Data Accessibility Statements

Data are available on reasonable request.

## Additional File

The additional file for this article can be found as follows:

10.5334/aogh.3278.s1 Appendices. Appendix 1 to 4.

## Abbreviations

AFEM: The African Federation for Emergency Medicine

AFEM-CMS: AFEM COVID-19 Mortality Score

AUROC: Area Under the Receiver Operating Curve

CFR: Case Fatality Rate

CI: Confidence Interval

COVID-19: Coronavirus Disease 2019

EU: Emergency Unit

GCS: Glasgow Coma Scale

HIC: High-Income Country

IQR: Interquartile Range

LASSO: Least Absolute Shrinkage and Selection Operator

LMICs: Low- and Middle-Income Countries

LRS: Low-Resource Setting

MICE: Multiple Imputation by Chained Equations

PCR: Polymerase Chain Reaction
