# Supplementary material for: Derivation of a Contextually-Appropriate COVID-19 Mortality Scale for Low-Resource Settings
Source: Ann Glob Health. 2021 Mar 26;87(1):31. doi: 10.5334/aogh.3278 (PMC7996452; doi:10.5334/aogh.3278)
Supplement: Appendices. — Appendix 1 to 4. [file agh-87-1-3278-s1.pdf]

Appendices

Appendix 1: Evidence for inclusion of candidate variables.

Variable	Evidence	Inclusion decision
**DEMOGRAPHICS**		
Age (years)	A-DROP,[1] APACHE II,[2] COVID-GRAM,[3] CURB-65,[4,5] MuLBSTA,[6] PSI,[4] SMART-COP,[7] 4C[5,8]	Included
Male sex at birth	A-DROP,[1] PSI,[4] 4C,[5,8] pOR: 1.35 (95% CI [1.21-1.50])[9]	Included
**COMORBIDITIES***		
Alcohol use	pOR: 1.56 (95% CI [0.70, 3.45])[9]	Included in composite "Total no. comorbidities" variable
Cardiovascular disease	PSI (congestive heart failure),[4] 4C (chronic cardiac disease)[5,8]	Included in composite "Total no. comorbidities" variable
Chronic respiratory disease	4C (chronic pulmonary disease, excluding asthma),[5,8] pOR for chronic obstructive pulmonary disease, asthma, interstitial lung disease, and pulmonary hypertension: 1.89 (95% CI [1.50, 2.38]);[9] pOR for tuberculosis: 2.68 (95% CI[0.91, 7.85])[9]	Included in composite "Total no. comorbidities" variable
Chronic neurological disease	PSI (cerebrovascular disease),[4] pOR for stroke: 3.08 (95% CI[1.95, 4.88])[9]	Included in composite "Total no. comorbidities" variable
Cirrhosis	PSI,[4] 4C (liver disease),[5,8] pOR: 1.94 (95% CI [1.07, 3.51])[9]	Included in composite "Total no. comorbidities" variable
Chronic kidney disease	PSI,[4] 4C,[5,8] pOR: 2·55 (95% CI [1·52-4·29])[9]	Included in composite "Total no. comorbidities" variable
Current or former smoker	MuLBSTA,[6] pOR: 1.49 (95% CI [1.17, 1.90])[9]	Included in composite "Total no. comorbidities" variable
Current pregnancy	No evidence identified	Excluded due to lack of evidence and excessive missingness in derivation dataset (65.4% missing for females)
Diabetes (types 1 and 2)	4C,[5,8] pOR: 1.72 (95% CI [1.45, 2.03])[9]	Included in composite "Total no. comorbidities" variable
Hypertension	MuLBSTA,[6] pOR: 2·14 (95% CI[1·82, 2·51])[9]	Included in composite "Total no. comorbidities" variable

Hypothyroid	COVID-GRAM,[3] pOR for thyroid disease: 1.58 (95% CI [0.46, 5.42])[9]	Included in composite "Total no. comorbidities" variable
Malignancy	PSI,[4] 4C,[5,8] pOR: 1.74 (95% CI[1.10, 2.74])[9]	Included in composite "Total no. comorbidities" variable
Rheumatological/connective tissue disease	No evidence identified	Excluded due to lack of evidence
Total no. comorbidities	COVID-GRAM,[3] 4C,[5,8] pOR: 2.41 (95% CI [2.01, 2.89])[9]	Included
**PRESENTING SIGNS & SYMPTOMS**		
Abdominal pain and/or distention	pOR: 1.58 (95% CI [1.12, 2.22])[9]	Included
Anorexia, nausea, or vomiting	pOR for anorexia: 1.61 (95% CI [1.26, 2.06]);[9] pOR for vomiting: 1.52 (95% CI [1.11, 2.10])[9]	Included
Anosmia	No evidence identified	Excluded due to lack of evidence
Chest pain or tightness	pOR for chest tightness: 1.62 (95% CI [1.11, 2.37]);[9] pOR for chest pain: 1.55 (95% CI [0.80, 3.01])[9]	Included
Convulsions	No evidence identified	Excluded due to lack of evidence
Cough	A-DROP,[1] pOR: 1.08 (95% CI [0.96, 1.23])[9]	Included
Diarrhoea	pOR: 1.10 (95% CI [0.95, 1.27])[9]	Included
Fatigue	pOR: 1.22 (95% CI [1.02, 1.45])[9]	Included
Fever	pOR: 1.22 (95% CI [1.08, 1.38])[9]	Included
Headache	No evidence identified	Excluded due to lack of evidence
Internal bleeding	COVID-GRAM,[3] pOR for haemoptysis: 1.51 (95% CI [0.82, 2.78])[9]	Included
Myalgia	No evidence identified	Excluded due to lack of evidence
Shortness of breath	COVID-GRAM,[3] pOR: 2.78 (95% CI [2.24, 3.46])[9]	Included
Sore throat	No evidence identified	Excluded due to lack of evidence
**VITAL SIGNS**		
Glasgow Coma Scale	APACHE II,[2] NEWS2,[10] PSI,[4] qSOFA,[5,8,11] 4C [5,8]	Included
Heart rate (beats/min)	APACHE II,[2] NEWS,[5,11] NEWS2,[10] PSI,[4] SIRS,[11] SMART-COP[7]	Included

Peripheral oxygen saturation	A-DROP,[1] BCRSS,[12,13] NEWS,[5,11] NEWS2,[10] qCSI,[14] 4C[5,8]	Included
Systolic blood pressure (mmHg)	CURB-65,[4,5] NEWS,[5,11] NEWS2,[10] PSI,[4] qSOFA,[5,8,11] SMART-COP[7]	Included
Respiratory rate (breaths/min)	APACHE II,[2] BCRSS,[12,13] CURB65,[4,5] NEWS,[5,11] NEWS2,[10] PSI,[4] qCSI,[14] qSOFA,[5,8,11] SIRS,[11] SMART-COP,[7] 4C[5,8]	Included
Temperature (°C)	APACHE II,[2] NEWS,[5,11] NEWS2,[10] PSI,[4] SIRS[11]	Excluded - excessive missingness in derivation dataset (86.7% missing)

Appendix 2. Variable selection using generalised additive models.[5]

Variable	Model for settings *with* access to pulse oximetry					Model for settings *without* access to pulse oximetry				
	Deviance explained (%)	Deviance explained (%)	Deviance explained (%)	Deviance explained (%)	Deviance explained (%)	Deviance explained (%)	Reduction in deviance explained on removal from full model (%)	R^2	Area under receiver operator curve	Inclusion in final model
**ALL CANDIDATE VARIABLES**	45	35.9	35.9	35.9	35.9	35.9	-	0.471	0.879	
Male sex at birth	44.3	34.7	34.7	34.7	34.7	34.7	0.7	0.466	0.877	Include
Age (years)	42.8	32.8	32.8	32.8	32.8	32.8	2.2	0.45	0.869	Include
Number of comorbidities*	43.5	34.4	34.4	34.4	34.4	34.4	1.5	0.456	0.873	Include
Current or former smoker	44.9	35.9	35.9	35.9	35.9	35.9	0.1	0.472	0.879	Exclude
Abdominal pain and/or distention	44.6	35.8	35.8	35.8	35.8	35.8	0.4	0.468	0.877	Exclude
Anorexia, nausea, and/or vomiting	44.6	35.8	35.8	35.8	35.8	35.8	0.4	0.468	0.877	Exclude
Chest pain or tightness	44.7	35.8	35.8	35.8	35.8	35.8	0.3	0.469	0.878	Exclude
Cough	44.6	35.7	35.7	35.7	35.7	35.7	0.4	0.469	0.876	Exclude
Diarrhoea	44.6	35.8	35.8	35.8	35.8	35.8	0.4	0.468	0.877	Exclude
Fatigue	44.7	35.9	35.9	35.9	35.9	35.9	0.3	0.471	0.878	Exclude
Internal bleeding**	44.9	35.9	35.9	35.9	35.9	35.9	0.1	0.473	0.879	Exclude
Shortness of breath	44.8	35.7	35.7	35.7	35.7	35.7	0.2	0.469	0.879	Exclude
Glasgow Coma Scale score	40.8	30.7	30.7	30.7	30.7	30.7	4.2	0.439	0.861	Include
Heart rate (beat/min)	44.6	33.9	33.9	33.9	33.9	33.9	0.4	0.466	0.876	Exclude
Systolic blood pressure (mmHg)	42.2	34.6	34.6	34.6	34.6	34.6	2.8	0.446	0.869	Include
Respiratory rate (breaths/min)	39.9	26.7	26.7	26.7	26.7	26.7	5.1	0.419	0.856	Include
Peripheral oxygen saturation (%)***	36	-	-	-	-	-	9	0.382	0.839	Include
**FINAL MODEL**	42.4	34.2	34.2	34.2	34.2	34.2	2.6	0.461	0.868	-

*Some comorbidities have been collapsed into the following clinically meaningful categories:

Cardiovascular disease includes one or more of the following: atrial fibrillation, congestive heart failure, coronary artery disease, deep vein thrombosis, dilated cardiomyopathy, ischaemic heart disease, myocardial infarction, and small vessel disease.

Chronic respiratory disease includes one or more of the following: asthma, chronic obstructive pulmonary disease, and tuberculosis.

Rheumatic/connective tissue disease includes one or more of the following: gout, lupus, and rheumatoid arthritis.

Chronic neurological disease includes one or more of the following: epilepsy, haemorrhagic or ischaemic stroke, and Parkinson's disease.

**Internal bleeding includes one or more of the following: gastrointestinal bleeding, haematuria, or haemoptysis.

***Peripheral oxygen saturation obtained on room air.

Appendix 3: Penalised regression coefficients from LASSO regression.

Variable	Level	Model for settings *with* access to pulse oximetry		Model for settings *without* access to pulse oximetry	
		**Penalised coefficient**	**Penalised coefficient**	**Penalised coefficient**	**Penalised coefficient**
Intercept	-	-0.157	-0.354	-0.354	-0.157
Sex at birth	Male	0.117	0.143	0.143	0.117
Age (years)	≥65	0.113	0.139	0.139	0.113
No. of comorbidities*	1	0.003	0.004	0.004	0.003
	≥2	0.088	0.098	0.098	0.088
Glasgow Coma Scale score	<15	0.233	0.276	0.276	0.233
Heart rate (beats/min)	>90	-	0.079	0.079	-
Systolic blood pressure (mmHg)	<100	0.112	0.238	0.238	0.112
Respiratory rate (breaths/min)	20-29	0.069	0.087	0.087	0.069
	≥30	0.076	0.092	0.092	0.076
Peripheral oxygen saturation (%)**	<92	0.304	-	-	0.304

*Comorbidities are defined as follows: Alcohol use, cardiovascular disease (one or more of the following: atrial fibrillation, congestive heart failure, coronary artery disease, deep vein thrombosis, dilated cardiomyopathy, ischaemic heart disease, myocardial infarction, and small vessel disease), chronic respiratory disease (one or more of the

following: asthma, chronic obstructive pulmonary disease, and tuberculosis), chronic neurological disease (one or more of the following: epilepsy, haemorrhagic or ischaemic stroke, and Parkinson's disease), cirrhosis, chronic kidney disease, current or former smoker status, diabetes (types 1 and 2), hypertension, hypothyroid, and malignancy.

**Peripheral oxygen saturation obtained on room air.

Appendix 4a: Comparison of AFEM COVID-19 Mortality Scale (AFEM-CMS) scores to case fatality rates in derivation cohort, for settings *with* access to pulse oximetry.

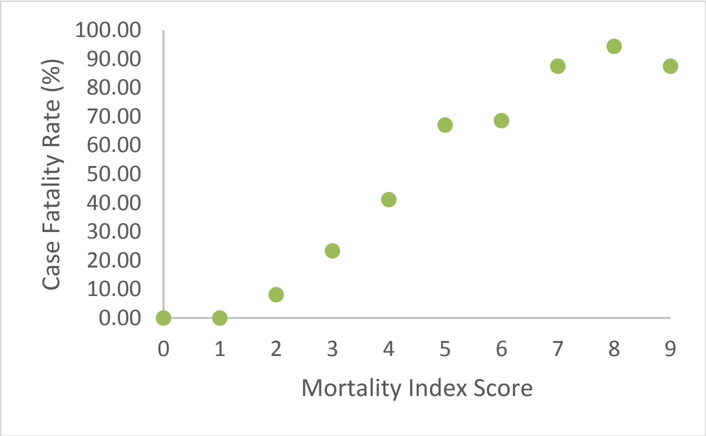

Appendix 4b: Comparison of AFEM COVID-19 Mortality Scale (AFEM-CMS) scores to case fatality rates in derivation cohort, for settings *without* access to pulse oximetry.

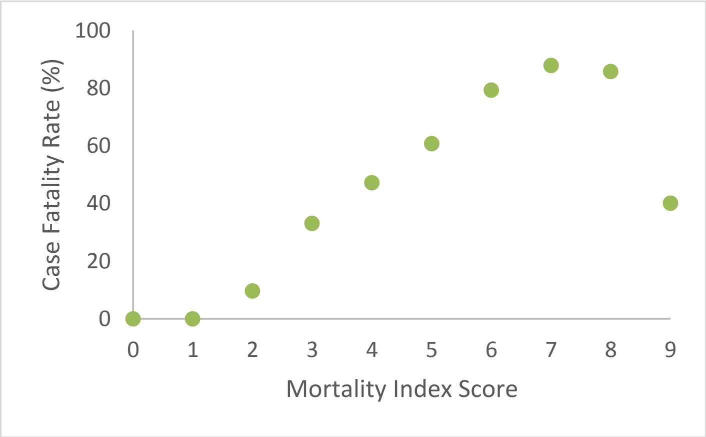

Appendices references

1. **Miyashita N, Matsushima T, Oka M, Japanese Respiratory S.** The JRS guidelines for the management of community-acquired pneumonia in adults: An update and new recommendations. Intern Med. 2006;45(7):419–428.
2. **Zou X, Li S, Fang M,** et al. Acute Physiology and Chronic Health Evaluation II Score as a Predictor of Hospital Mortality in Patients of Coronavirus Disease 2019. Crit Care Med. 2020;48(8):e657–e665.
3. **Liang W, Liang H, Ou L,** et al. Development and Validation of a Clinical Risk Score to Predict the Occurrence of Critical Illness in Hospitalized Patients With COVID-19. JAMA Intern Med. 2020;180(8):1081–1089.
4. **Satici C, Demirkol MA, Sargin Altunok E,** et al. Performance of pneumonia severity index and CURB-65 in predicting 30-day mortality in patients with COVID-19. Int J Infect Dis. 2020;98:84–89.
5. **Wellbelove Z, Walsh C, Perinpanathan T, Lillie P, Barlow G.** Comparing the 4C mortality score for COVID-19 to established scores (CURB65, CRB65, qSOFA, NEWS) for respiratory infection patients. J Infect. 2020.
6. **Iijima Y, Okamoto T, Shirai T,** et al. MuLBSTA score is a useful tool for predicting COVID-19 disease behavior. J Infect Chemother. 2020.
7. **Fan G, Tu C, Zhou F,** et al. Comparison of severity scores for COVID-19 patients with pneumonia: A retrospective study. Eur Respir J. 2020;56(3).
8. **Knight SR, Ho A, Pius R,** et al. Risk stratification of patients admitted to hospital with covid-19 using the ISARIC WHO Clinical Characterisation Protocol: Development and validation of the 4C Mortality Score. BMJ. 2020;370:m3339.
9. **Pigoga J, Friedman A, Broccoli M,** et al. Clinical and historical features associated with severe COVID-19 infection: A systematic review and meta-analysis. Paired paper submission. 2020.

10. **Myrstad M, Ihle-Hansen H, Tveita AA,** et al. National Early Warning Score 2 (NEWS2) on admission predicts severe disease and in-hospital mortality from Covid-19 - a prospective cohort study. Scand J Trauma Resusc Emerg Med. 2020;28(1):66.

11. **Jang JG, Hur J, Hong KS, Lee W, Ahn JH.** Prognostic Accuracy of the SIRS, qSOFA, and NEWS for Early Detection of Clinical Deterioration in SARS-CoV-2 Infected Patients. J Korean Med Sci. 2020;35(25):e234.

12. **Duca A, Piva S, Foca E, Latronico N, Rizzi M.** Calculated Decisions: Brescia-COVID Respiratory Severity Scale (BCRSS)/Algorithm. Emerg Med Pract. 2020;22(5 Suppl):CD1-CD2.

13. **Rodriguez-Nava G, Yanez-Bello MA, Trelles-Garcia DP, Chung CW, Friedman HJ, Hines DW.** Performance of the Quick COVID-19 Severity Index and the Brescia-COVID Respiratory Severity Scale in hospitalized patients with COVID-19 in a community hospital setting. Int J Infect Dis. 2020.

14. **Haimovich AD, Ravindra NG, Stoytchev S,** et al. Development and Validation of the Quick COVID-19 Severity Index: A Prognostic Tool for Early Clinical Decompensation. Ann Emerg Med. 2020;76(4):442–453.
